# Dosimetric comparison of various optimization techniques for high dose rate brachytherapy of interstitial cervix implants

**DOI:** 10.1120/jacmp.v11i3.3227

**Published:** 2010-07-12

**Authors:** Bondel Shwetha, Manickam Ravikumar, Aradhana Katke, Sanjay S. Supe, Golhalli VenkataGiri, Nanda Ramanand, Tanvir Pasha

**Affiliations:** ^1^ Department of Radiation Physics Kidwai Memorial Institute of Oncology Karnataka India; ^2^ Department of Radiotherapy Kidwai Memorial Institute of Oncology Karnataka India

**Keywords:** optimization, high dose rate, interstitial implant, target coverage, isodose reshape

## Abstract

HDR brachytherapy treatment planning often involves optimization methods to calculate the dwell times and dwell positions of the radioactive source along specified afterloading catheters. The purpose of this study is to compare the dose distribution obtained with geometric optimization (GO) and volume optimization (VO) combined with isodose reshaping. This is a retrospective study of 10 cervix HDR interstitial brachytherapy implants planned using geometric optimization and treated with a dose of 6 Gy per fraction. Four treatment optimization plans were compared: geometric optimization (GO), volume optimization (VO), geometric optimization followed by isodose reshape (GO_IsoR), and volume optimization followed by isodose reshape (VO_IsoR). Dose volume histogram (DVH) was analyzed and the four plans were evaluated based on the dosimetric parameters: target coverage ($V_{100}$), conformal index (COIN), homogeneity index (HI), dose nonuniformity ratio (DNR) and natural dose ratio (NDR). Good target coverage by the prescription dose was achieved with GO_IsoR (mean $V_{100}$ of 88.11%), with 150% and 200% of the target volume receiving 32.0% and 10.4% of prescription dose, respectively. Slightly lower target coverage was achieved with VO_IsoR plans (mean $V_{100}$ of 86.11%) with a significant reduction in the tumor volume receiving high dose (mean $V_{150}$ of 28.29% and mean $V_{200}$ of 7.3%). Conformity and homogeneity were good with VO_IsoR (mean COIN=0.75 and mean HI=0.58) as compared to the other optimization techniques. VO_IsoR plans are superior in sparing the normal structures while also providing better conformity and homogeneity to the target. Clinically acceptable plans can be obtained by isodose reshaping provided the isodose lines are dragged carefully.

PACS number: 87.53 Bn

## INTRODUCTION

High dose rate (HDR) interstitial brachytherapy is a major treatment option for localized cervical carcinoma. HDR interstitial brachytherapy for patients with advanced cervical carcinoma has been reported.^(^ ^1^ ^)^ A high rate of pelvic control and survival with acceptable level of late toxicities were obtained for patients with previously untreated cervical carcinoma in which intracavitary brachytherapy may result in a suboptimal dose distribution. HDR brachytherapy treatment planning often involves optimization methods to calculate the dwell times and dwell positions of the radioactive source along specified applicator paths. The goal of HDR planning is to produce an acceptable optimized plan within a reasonable time period, which meets the desired dose constraints. Optimization codes for interstitial implants aim at obtaining adequate target coverage and maximum sparing of critical structures.

The effects of geometric optimization in a regular and irregular volume implant have been analyzed by Kolkman‐Deurloo et al. ^(^ ^2^ ^)^ In geometric optimization, the relative dwell times are determined by the geometry of the implant by assigning an individual weighting factor for the dwell time at each dwell position that is inversely proportional to the dose contribution from neighboring source locations. Geometric optimization was concluded to be an effective tool to improve the dose distribution of interstitial volume implants. A quantitative assessment of interstitial implants is proposed by Saw and Suntharalingam^(^ ^3^ ^)^ using volume versus dose curves and four dosimetric parameters namely, dose nonuniformity ratio (DNR), coverage index (CI), external volume index (EI) and relative dose homogeneity index (HI). The differential and cumulative volume versus dose curves provides quantitative data on the volume of tissues irradiated to different doses and qualitative assessment of the variations in dose delivery.

Recently, a comparison of anatomy‐based inverse planning with simulated annealing (IPSA) and graphical optimization for high dose rate prostate brachytherapy was reported by Morton et al.^(^ ^4^ ^)^ They observed that plans generated using IPSA provide similar dose coverage to target compared to those obtained using graphical optimization. IPSA plans delivered lower dose to normal structures and greater dose homogeneity than graphical optimization plans. It has been reported by Jamema et al.^(^ ^5^ ^)^ that anatomy based inverse optimization followed by graphical optimization is much superior in conformity and sparing of critical structures as compared to geometric optimization. Akimoto et al.^(^ ^6^ ^)^ have evaluated the advantages of anatomy‐based inverse optimization (ABIO) over geometric optimization (GO) in HDR brachytherapy of prostate cancer. They reported lower dose to urethra and significantly lower acute genitourinary toxicities in patients planned using ABIO when compared to GO.

At our institute, BrachyVision version 6.5 treatment planning system (Varian Medical Systems, Inc., Palo Alto, CA) has been installed recently. The various optimization routines available in BrachyVision ^(^ ^7^ ^)^ are: (i) equal times, which optimizes using equal dwell times along applicators, (ii) geometric optimization (GO), where the dwell times are adjusted to produce a uniform dose around the applicators performed by normalizing the dose to a reference point within the target, and (iii) volume optimization (VO), a method of inverse planning which attempts to deliver a specified dose to a particular or to many anatomical structures. Apart from these techniques, the dose distribution can also be altered by using the “isodose reshaping” (IsoR) tool which allows the planner to manually drag the isodose lines on each individual CT slice on the screen using the mouse. The IsoR algorithm then recalculates the dwell times according to the preselected isodose curves.

In our institute, routine high dose rate interstitial brachytherapy treatment planning is performed using the geometric optimization procedure. In the present study, a comparison has been made between the dose distributions obtained using geometric and volume optimization techniques followed by isodose reshaping for HDR cervix interstitial implants.

## II. MATERIALS AND METHODS

This is a study of 10 patients with cervical carcinoma treated with HDR interstitial brachytherapy. The patients selected for the study were of stage IIIB (6 out of 10) and stage IIB (4 out of 10). The interstitial implant was performed using Syed‐Neblett gynecologic template. An average of 20 needles was used for the implant. Post‐implant CT scans of 5 mm slice thickness were obtained. Treatment planning was performed using BrachyVision version 6.5 treatment planning system. The catheters were reconstructed and anatomic structures (target, rectum and bladder) were delineated. All patient treatments were planned using GO. The entire bladder was contoured. The prescribed dose was 6 Gy for each patient. Dose reference point is placed in the target volume and normalization performed on the point to obtain the dose distribution. The GO was followed by isodose reshaping done by manual adjustments of isodose lines using the mouse. The reference isodose is that isodose which conforms to the target adequately. While adjusting the isodose lines, care was taken to limit the dose to the critical structures within their tolerance limits.

Volume optimization requires constraints for target and critical structures as input. The dose constraints were defined through a trial and error process. The minimum and maximum dose limits to the target were 95% and 120%, respectively (priority 100% and above). The maximum dose to bladder and rectum was set at 60% of prescribed dose. VO was then followed by isodose reshaping.

Dose volume histograms of target, bladder and rectum were evaluated. The following dosimetric data were collected for the four optimization techniques: $V_{100}$, $V_{150}$, $V_{200}$ for the target, maximum dose to 5 cc of bladder and rectum, $V_{80}$ and $V_{100}$ for bladder and rectum. The conformal index (COIN), relative dose homogeneity index (HI), and dose nonuniformity ratio (DNR) were computed from cumulative DVHs, and the natural dose ratio (NDR) from natural DVH. COIN ^(^ ^8^ ^)^ describes how well the reference isodose encompasses the target volume and excludes non‐target structures.

(1) $$\text{COIN}=c_{1}\times c_{2}\times c_{3}$$

where $c_{1}$ is the fraction of PTV that is enclosed by reference isodose, $c_{2}$ is the fraction of the reference isodose volume ${(V}_{\text{ref}})$ that is covered by PTV, $c_{3}$ is a term which accounts for the unwanted irradiation of parts or all of a critical structure which can be determined by the expression:

(2) $$c_{3}=\prod_{i=1}^{N_{\text{CS}}}\left[1-V_{\text{CSref},I}/V_{\text{CS},I}\right]$$

where $N_{\text{CS}}$ is the total number of critical structures, $V_{\text{CSref}}$ is the volume of critical structure encompassed by reference isodose line, and $V_{\text{CS}}$ is the total volume of the critical structure.

HI ^(^ ^9^ ^)^ measures the fraction of target volume receiving dose in the interval of 1.0 to 1.5 times the reference dose.

(3) $$\text{HI}=(V_{100}-V_{150})/V_{\text{tot}}$$

where $V_{100}$ and $V_{150}$ are the volumes of PTV receiving 100% and 150% of the prescription dose, respectively, and $V_{\text{tot}}$ is the total target volume.

${\text{DNR}}^{\left(3,10\right)}$ is defined as the ratio of high‐dose volume relative to the reference volume.

(4) $$\text{DNR}=V_{150}/V_{100}$$

NDR ^(^ ^11^ ^)^ is a means of quantifying the degree of underdosage or overdosage of a particular implant.

(5) NDR=NPD/PD

where NPD and PD are the natural prescription dose and actual prescription dose of the implant, respectively. NPD is located at the base of the peak of natural dose volume histogram (NDVH).

For an ideal implant the position of the prescription dose (PD) is at the base of the peak, so that the peak dose area of the implant lies inside the prescription isodose surface and the area of dose decrease lies outside it. This optimal PD is called natural prescription dose (NPD) because it can only be determined by the NDVH.

## III. RESULTS

**Figure 1:**
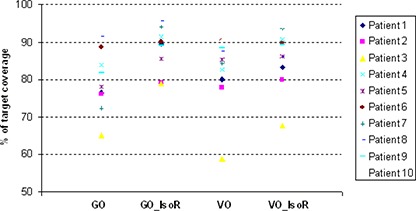
Graphical representation of volume of target receiving 100% dose with the four optimization techniques.

Dosimetric parameters were tabulated for GO, GO followed by isodose reshape (GO_IsoR), VO and VO followed by isodose reshape (VO_IsoR) in Table 1. Good target coverage by the prescription dose was achieved with GO_IsoR (mean $V_{100}$ of 88.11%) with 150% and 200% of the target volume receiving 32.0% and 10.4% of prescription dose, respectively. Slightly lower target coverage was achieved with VO_IsoR plans (mean $V_{100}$ of 86.11%) with a significant reduction in the tumor volume receiving high dose (mean $V_{150}$ of 28.3% and mean $V_{200}$ of 7.3%, respectively). Figure 1 shows the plot of target coverage for the four optimization techniques.

**Table 1:** Dosimetric parameters for the different optimization techniques.

*Parameter*	*GO Mean (Std Dev)*	*GO_IsoR Mean (Std Dev)*	*VO Mean (Std Dev)*	*VO_IsoR Mean (Std Dev)*
Target $V_{100}$	79.97(8.0)%	88.11(5.6)%	82.53(9.3)%	86.11(7.8)%
Target $V_{150}$	31.16(11.1)%	32.00(6.1)%	27.98(8.4)%	28.29(5.2)%
Target $V_{200}$	6.74(4.4)%	10.38(5.5)%	9.38(4.4)%	7.30(4.3)%
COIN	0.61(0.05)	0.73(0.06)	0.69(0.08)	0.75(0.10)
HI	0.49(0.10)	0.56(0.08)	0.55(0.11)	0.58(0.10)
NDR	1.04(0.06)	0.92(0.07)	0.88(0.08)	0.94(0.09)
DNR	0.39(0.12)	0.36(0.07)	0.34(0.10)	0.33(0.08)
Bladder $D_{5\text{cc}}$	78.53(22.6)%	72.68(16.9)%	75.33(24.2)%	69.62(15.1)%
Bladder $V_{80}$	9.11(8.3)%	6.24(5.0)%	6.40(6.5)%	4.82(4.6)%
Bladder $V_{100}$	3.13(3.7)%	0.66(0.59)%	1.84(2.7)%	0.46(0.81)%
Rectum $D_{5\text{cc}}$	80.28(9.4)%	76.39(6.5)%	77.33(10.1)%	74.71(6.5)%
Rectum $V_{80}$	8.60(4.9)%	5.99(3.2)%	6.01(3.4)%	5.24(3.0)%
Rectum $V_{100}$	2.36(1.9)%	0.56(1.0)%	0.91(0.67)%	0.34(0.50)%

GO=geometric optimization, GO_IsoR=geometric optimization followed by isodose reshape, VO=volume optimization, VO_IsoR=volume optimization followed by isodose reshape, COIN=conformal index, HI=homogeneity index, NDR=natural dose ratio, DNR=dose nonuniformity ratio, $V_{1000}=\text{volume}$ receiving 100% of prescription dose, $D_{5\text{cc}}=\text{dose}$ received by 5 cc of the volume.

**Figure 2:**
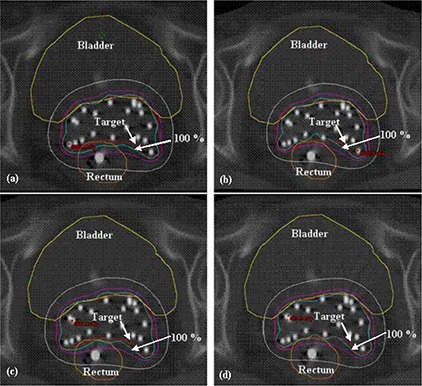
Isodose distribution on the same CT slice for (a) GO; (b) GO_IsoR; (c) VO; (d) VO_IsoR.

The COIN describes how well the reference isodose line conforms to the target volume and excludes the surrounding normal structures. Conformity was good with VO_IsoR as compared to the other optimization techniques (mean COIN of 0.75). The mean homogeneity index values are 0.49, 0.56, 0.55 and 0.58 for GO, GO_IsoR, VO and VO_IsoR, respectively. Homogeneity was good with VO_IsoR as compared to other techniques. The natural dose ratio was nearer to 1.0 with GO and VO_IsoR (1.04 and 0.94, respectively), which shows that there is slight overdosage and underdosage in these two techniques compared to other techniques. The dose nonuniformity ratio was minimum with VO_IsoR (mean DNR of 0.33). Mean doses to bladder and rectum were least with VO_IsoR (mean $V_{80}$ of 4.82% and 5.24%, $V_{100}$ of 0.46% and 0.34%, and $D_{5\text{cc}}$ of 69.62% and 74.71% for bladder and rectum, respectively). For the same implant case, the CT images with dose distribution using the four optimization techniques are shown in Fig. 2. It can be observed that bladder and rectum are spared to the maximum extent with VO_IsoR while not compromising with the target coverage. GO_IsoR also spares the critical organs but with reduced target homogeneity as compared to VO_IsoR.

## IV. DISCUSSION

In case of VO, target coverage is sometimes reduced when trying to satisfy the critical structure constraints. In that case, the priority of the target volume was increased in order to obtain better conformity. It was observed that volume optimization provides good target coverage while also reducing dose to the critical structures. The dose to bladder and rectum was higher with GO while it was least with VO_IsoR. VO followed by isodose reshape fine tunes the distribution in terms of conformity, homogeneity, target coverage and critical organ sparing. Isodose reshaping is a useful tool that allows the planner to drag the isodose lines on the screen as desired. Care should be taken to see that, while adjusting the isodose line in one CT slice, dose distribution in the adjacent slices are not altered significantly. This tool should be used only for fine‐tuning the optimized dose distribution. Clinically acceptable plans can be obtained by isodose reshaping, provided the isodose lines are dragged carefully. In the present study, utmost care was taken while reshaping the isodose lines to ensure that the target coverage was optimum while at the same time restricting the doses to bladder and rectum to as low as possible.

## V. CONCLUSIONS

Considering the overall parameters, VO_IsoR plans are superior compared to the plans obtained with other optimization techniques in sparing the normal structures while also providing better conformity and homogeneity to the target. Though treatment planning using isodose reshaping is a time consuming process, with greater experience of the planner dose optimization can be performed within a smaller time frame using this tool. Hence, the dose distribution can be varied as desired and clinically acceptable plans can be obtained using volume optimization combined with isodose reshaping.
